# An IoT-Edge Enabled Deep–Fuzzy Hybrid Model for Real-Time Indoor Air Quality Optimization

**DOI:** 10.3390/s26133989

**Published:** 2026-06-23

**Authors:** Samia Allaoua Chelloug, Mohammed Muthanna, Abdullah Alshahrani, Mohammad Hassan Ali Al-Onaizan, Ammar Muthanna, Faisal Jamil

**Affiliations:** 1 Department of Information Technology, College of Computer and Information Sciences, Princess Nourah Bint Abdulrahman University, Riyadh 11671, Saudi Arabia; sachelloug@pnu.edu.sa; 2 Faculty of Computing and Information Technology, Sohar University, Sohar 311, Oman; mmuthanna@su.edu.om; 3 Department of Computer Science and Artificial Intelligence, College of Computer Science and Engineering, University of Jeddah, Jeddah 21493, Saudi Arabia; asalshahrani2@uj.edu.sa; 4 Department of Intelligent Systems Engineering, Faculty of Engineering and Design, Middle East University, Amman 11831, Jordan; m.alonaizan@meu.edu.jo; 5 Institute of Computer Science and Telecommunications, RUDN University, 117198 Moscow, Russia; mutkhanna-as@rudn.ru; 6 Department of AI and Software, Gachon University, Seongnam 13120, Republic of Korea

**Keywords:** indoor air quality, Comfort Risk Index, knowledge distillation, fault tolerance, human-centric control, edge computing

## Abstract

Indoor air quality has a significant impact on occupant health, comfort, and productivity in residential and commercial indoor environments. This paper proposes an IoT-edge enabled deep–fuzzy hybrid framework for real-time IAQ prediction and adaptive control. The proposed system integrates IoT-based environmental sensing, Temporal Fusion Transformer-based multivariate forecasting, knowledge distillation, edge-deployed Bi-LSTM inference, and Mamdani fuzzy logic control within a unified IAQ management architecture. A composite Comfort Risk Index is introduced to combine environmental parameters and occupant discomfort feedback into a single adaptive control indicator. Experimental evaluation under varying indoor conditions demonstrated strong forecasting performance, with prediction accuracies reaching 96.3% for CO_2_ and 95.7% for PM_2.5_ prediction, while reducing inference latency from 575 ms to 295 ms. Comparative analysis against baseline threshold-based control strategies further indicated improved comfort stability, smoother actuator behavior, and reduced estimated actuator operating intensity during deployment. The proposed framework also demonstrated resilient operation under simulated sensor-failure conditions while maintaining low computational overhead suitable for resource-constrained IoT-edge environments. Overall, the results indicate that combining lightweight deep learning models with interpretable fuzzy control can provide an effective, scalable, and energy-aware solution for intelligent real-time IAQ optimization in smart indoor environments.

## 1. Introduction

Indoor air quality (IAQ) has become a critical public health and smart-building challenge due to rapid urbanization, energy-efficient building practices, and increasing indoor occupancy density [1]. Since individuals spend a significant portion of their time indoors, exposure to pollutants such as CO_2_, PM_2.5_, volatile organic compounds, and humidity imbalance can negatively affect respiratory health, cognitive performance, and overall well-being [2]. Recent concerns regarding airborne disease transmission, sustainable building management, and energy-aware environmental regulation have further increased the need for intelligent IAQ monitoring and adaptive control systems capable of operating under dynamic indoor conditions [3].

To address these challenges, advances in IoT sensing, wireless communication, and edge computing technologies have enabled continuous real-time monitoring of indoor environments using distributed low-cost sensor networks [4,5]. However, transforming continuous environmental sensing data into efficient and adaptive control actions remains a difficult task. Conventional IAQ control strategies are typically threshold-based or rule-based and often fail to capture complex multivariate environmental relationships, resulting in delayed or sub-optimal responses under changing indoor conditions [6]. Although recent data-driven forecasting approaches have demonstrated strong predictive performance, many existing methods remain computationally expensive, difficult to interpret, and challenging to deploy in resource-constrained edge environments [7].

Indoor environmental conditions are inherently dynamic and are strongly influenced by occupant activity, ventilation behavior, outdoor environmental changes, thermal conditions, and pollutant accumulation patterns [8]. Variations in CO_2_, particulate matter, humidity, and temperature are often interdependent and may change rapidly over time under different occupancy and ventilation scenarios [9,10]. As a result, maintaining acceptable IAQ requires continuous multivariate monitoring and adaptive environmental control rather than static threshold-based operation. Traditional ventilation and HVAC control strategies frequently respond reactively after pollutant concentrations exceed predefined limits, which may lead to delayed intervention, unnecessary energy consumption, and reduced occupant comfort [11].

Motivated by these limitations, recent research has increasingly explored statistical forecasting, deep learning, fuzzy control, and edge intelligence techniques for IAQ prediction and adaptive environmental management. Statistical models such as ARIMA and Prophet provide baseline forecasting capability but remain limited for highly dynamic multivariate IAQ data. Deep learning architectures including LSTM, GRU, CNN-LSTM, and Temporal Fusion Transformer (TFT) models have demonstrated improved temporal modeling of pollutant and comfort indicators. IndoAirSense [12] combines low-cost sensing with MLP, XGBR, and LSTM-wf models for classroom IAQ prediction, while [13] presents smartphone-linked CO_2_ forecasting using wireless sensing and machine learning. In addition, recent review studies [14,15] highlight persistent limitations related to adaptability, occupancy awareness, interpretability, and deployment scalability in existing IAQ forecasting systems.

Beyond forecasting, several studies have investigated intelligent environmental control and optimization strategies to improve comfort regulation and energy-aware operation [16]. Wang et al. [17] proposed a CFD-informed CNN-LSTM framework for thermal comfort prediction, while adaptive ARX-based IAQ prediction with occupancy integration was investigated in [18]. Other studies have incorporated fuzzy reasoning, neural networks, reinforcement learning, and swarm optimization for adaptive HVAC and environmental control [19,20,21]. At the same time, edge AI and TinyML deployment strategies have become increasingly important for enabling low-latency inference and scalable real-time environmental control in resource-constrained IoT systems [22,23,24,25]. Despite these advances, existing IAQ systems still face several important limitations. Many existing approaches prioritize either predictive accuracy or control optimization while overlooking interpretability, fault tolerance, occupant feedback integration, and deployment scalability under real-world operating conditions. Furthermore, several deep learning approaches remain unsuitable for lightweight edge deployment because of latency and computational overhead constraints. Existing control architectures also often lack human-centric adaptability and robust fallback mechanisms under partial sensor observability or sensor degradation.

To address these research gaps, this paper proposes an IoT-edge enabled deep–fuzzy hybrid framework for real-time IAQ prediction and adaptive control. The proposed architecture integrates multivariate forecasting, knowledge distillation, edge intelligence, and interpretable fuzzy reasoning within a unified IAQ management framework. A cloud-trained Temporal Fusion Transformer (TFT) model is employed to learn complex temporal dependencies, while knowledge distillation is used to generate a lightweight Bi-LSTM student model suitable for low-latency edge deployment. Forecasted environmental indicators are combined into a composite Comfort Risk Index (CRI), which is further processed through a Mamdani fuzzy inference system to generate adaptive ventilation, purification, and HVAC control actions. The framework additionally incorporates chatbot-assisted occupant feedback and fallback reasoning mechanisms to improve human-centric adaptability and resilience under dynamic indoor conditions.

The main contributions of this work are summarized as follows:

- An IoT-edge enabled deep–fuzzy hybrid architecture integrating multivariate IAQ forecasting and interpretable fuzzy control for adaptive real-time indoor environmental management.
- A teacher–student forecasting framework based on cloud-trained TFT models and lightweight Bi-LSTM edge inference through knowledge distillation for low-latency deployment.
- A composite CRI framework integrating environmental indicators and occupant discomfort feedback into a unified comfort-aware control representation.
- A Mamdani fuzzy inference system for context-aware adaptive control of ventilation, purification, and HVAC operation using interpretable rule-based reasoning.
- Comprehensive experimental evaluation in terms of forecasting performance, control responsiveness, fault resilience, interpretability, and deployment suitability under heterogeneous indoor environmental conditions.

Despite recent progress in IAQ forecasting, intelligent environmental control, and edge-enabled deployment, several important limitations remain unresolved, as summarized in Table 1. Existing studies primarily focus on either predictive accuracy or environmental control optimization, while limited attention has been given to the integration of forecasting, interpretable control, lightweight deployment, and occupant-centric adaptability within a unified framework. In particular, many deep-learning-based approaches achieve strong forecasting performance but remain computationally expensive for real-time edge deployment and often lack transparent decision-making mechanisms. Similarly, several optimization-driven HVAC and IAQ control systems improve energy efficiency and comfort regulation but provide limited fault resilience, weak interpretability, and insufficient integration with real-time multivariate forecasting models. Existing edge intelligence studies additionally focus mainly on deployment efficiency and communication optimization without incorporating adaptive environmental reasoning or human-in-the-loop feedback mechanisms. These limitations indicate the need for an integrated IAQ management framework capable of combining accurate multivariate forecasting, lightweight edge intelligence, interpretable fuzzy reasoning, fault resilience, and adaptive occupant-aware control within a single architecture. Motivated by these research gaps, the proposed framework integrates knowledge-distilled forecasting, CRI-driven fuzzy inference, chatbot-assisted occupant feedback, and adaptive edge deployment to enable robust, low-latency, and human-centric IAQ optimization under dynamic indoor conditions.

The remainder of this paper is organized as follows: Section 2 presents the proposed methodology, including preprocessing, forecasting, CRI formulation, and fuzzy control design. Section 3 describes the experimental setup and evaluation metrics. Section 4 presents the results and discussion, while Section 5 concludes this paper and outlines future research directions.

## 2. Proposed Deep–Fuzzy Hybrid IAQ Framework

**Figure 1:**
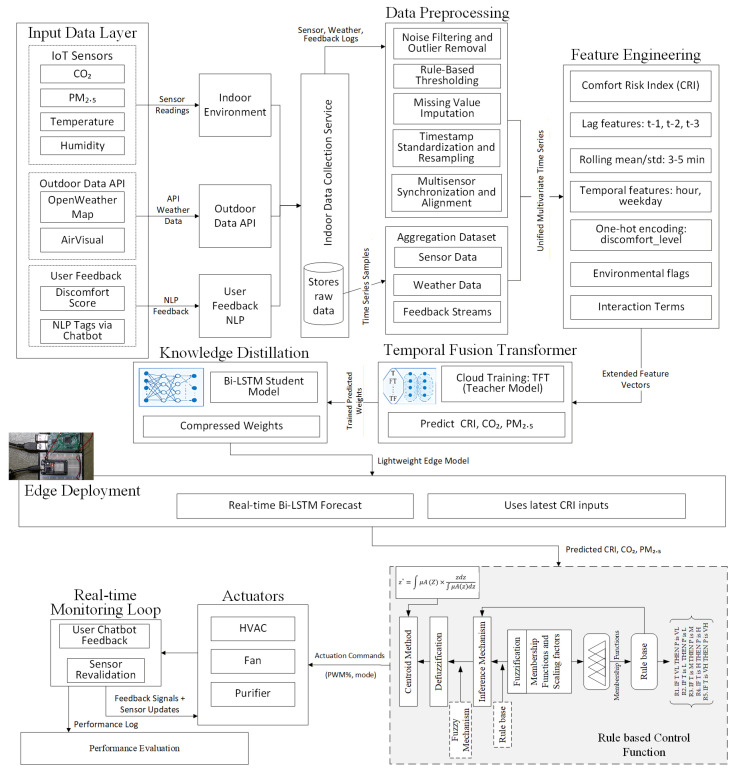
The proposed architecture of intelligent indoor air quality optimization.

The proposed deep–fuzzy hybrid framework is designed for real-time IAQ optimization through the integration of heterogeneous data sources, such as IoT sensor readings, environmental APIs, and user feedback into a multilevel pipeline that facilitates multivariate forecasting and adaptive actuator control. The system architecture, depicted in Figure 1, consists of data acquisition, preprocessing, deep model training, fuzzy control, and edge deployment modules. The forecasting component employs a cloud-trained TFT to acquire intricate temporal connections between environmental factors. A composite CRI integrates sensor measurements and user-reported discomfort to support adaptive control decisions. A Mamdani fuzzy inference system forecasts and processes this CRI to generate context sensitive actuation strategies. To enable low-latency inference in practical deployments, the TFT model is distilled into a lightweight Bi-LSTM student model suitable for edge execution.

The end-to-end framework enables continuous monitoring of indoor environmental conditions, real-time occupant comfort assessment, and adaptive control actions in dynamic indoor environments. The pipeline starts with the acquisition of real-time data using IoT sensors, weather APIs, and chatbot-based user feedback, followed by data cleaning, validation, noise reduction, and outlier removal. The heterogeneous streams of data are then aligned and resampled in time to assure uniformity and finally the features to be used in the context are designed to detect pertinent patterns of the environment and the user. These characteristics are standardized and coded and then utilized to train the TFT model in the cloud to predict in multiple steps. Knowledge distillation is then utilized to extract a lightweight model of Bi-LSTM student to be run on edges. A prediction result is combined with a Mamdani fuzzy logic controller so that IAQ systems can be controlled in real time and context-dependent. Lastly, the framework is tested using forecasting accuracy, control responsiveness and user-friendly comfort measures to confirm its usefulness in a variety of indoor settings.

### 2.1. Real-Time IAQ Optimization and Control

The practical objective of the proposed framework is to maintain indoor air quality within acceptable comfort and health-oriented operating conditions under dynamic indoor environments. In real-world indoor spaces, pollutant concentration and thermal comfort conditions continuously vary due to occupant activity, ventilation behavior, outdoor environmental changes, and equipment operation. As a result, maintaining stable indoor air quality requires continuous monitoring and adaptive control of environmental variables rather than static threshold-based regulation. In the proposed framework, the controlled variables include CO_2_, PM_2.5_, humidity, temperature, and the derived CRI, while the manipulated control variables include ventilation level, fan speed, air purifier intensity, and HVAC operating mode. The practical control objective is to minimize pollutant accumulation and discomfort risk while avoiding excessive actuator operation and unnecessary energy consumption. The proposed optimization framework operates by continuously predicting short-term indoor environmental conditions using multivariate time-series prediction models deployed within the IoT-edge architecture. The predicted environmental states are processed through the Mamdani fuzzy inference engine to determine adaptive actuator responses in real time. For example, elevated predicted CO_2_ and CRI levels increase ventilation and fan intensity, while high PM_2.5_ levels activate adaptive purifier operation. Unlike conventional reactive HVAC systems that respond only after pollutant concentrations exceed predefined thresholds, the proposed framework performs proactive and gradual control adjustment based on predicted environmental trends and occupant discomfort conditions.

### 2.2. Exploratory Data Analysis and Preprocessing

The dataset used in this research was collected using an IoT-based real-time indoor air-quality-monitoring system deployed within a smart-building testbed. The collected data consist of indoor environmental sensor time-series measurements, external weather API information, and occupant discomfort feedback collected through a chatbot interface. The experimental deployment was conducted across multiple representative indoor zones within a smart-building testbed environment, including living room, bedroom, kitchen, office-style workspace, and enclosed utility-space conditions. These deployment zones were selected to emulate heterogeneous residential and small-office indoor environments with varying occupancy behavior, ventilation conditions, and pollutant generation patterns. The monitored spaces represented small-to-medium indoor areas with variable occupant density ranging from low-occupancy single-user conditions to moderate multi-occupant activity scenarios during the 30-day monitoring period. Both naturally ventilated and HVAC-assisted operating conditions were included to evaluate the robustness of the proposed framework under diverse real-world indoor environmental dynamics.

**Figure 2:**
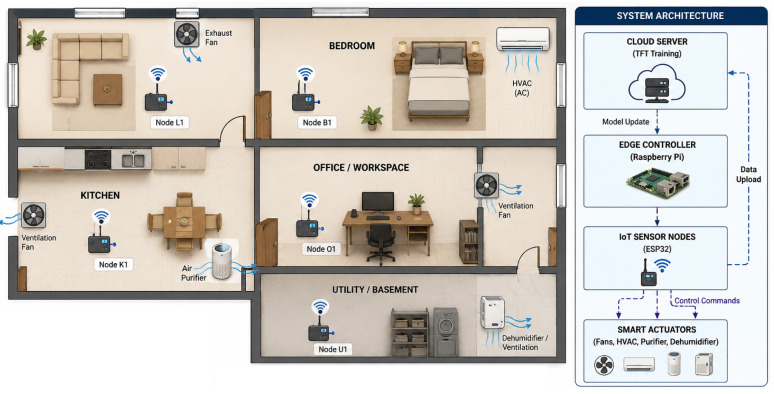
Smart-building IAQ deployment layout showing monitored indoor zones, IoT sensing nodes, adaptive control components, and integrated environmental sensors per node including CO_2_, PM_2.5_, temperature, humidity, and TVOC modules.

Figure 2 illustrates the representative indoor deployment layout used during data collection and experimental validation. The monitoring setup consisted of multiple indoor zones with heterogeneous occupancy behavior, ventilation conditions, and pollutant generation characteristics. IoT sensing nodes and smart actuators were distributed across the monitored spaces to support continuous environmental monitoring and adaptive IAQ control under realistic indoor operating conditions.

Indoor measurements included PM_2.5_, PM_10_, CO_2_, temperature, and humidity collected using PMS7003, SCD30, and DHT22 sensors connected to ESP32-based IoT nodes, while the SDS011 sensor was used for outdoor particulate matter monitoring. Sensor readings were recorded at 5 s intervals, while outdoor environmental variables including wind speed, pressure, temperature, and AQI were retrieved every 10 min using OpenWeatherMap and AirVisual APIs. User discomfort feedback was periodically collected and time-stamped using UTC. Sensors were installed at approximate breathing-zone height and positioned away from direct airflow paths, windows, doors, and HVAC outlets to reduce localized measurement bias and improve consistency across monitoring zones. Prior to deployment, all sensing devices underwent calibration and validation procedures to improve measurement reliability and consistency. The SCD30 CO_2_ sensors were subjected to automatic baseline calibration (ABC) and verified under outdoor fresh-air conditions (approximately 400–420 ppm CO_2_) to confirm baseline response. PMS7003 and SDS011 particulate matter sensors were co-located for 48 h and cross-compared to identify systematic offsets and sensor-to-sensor variability. Temperature and humidity measurements were assessed through side-by-side comparison of deployed sensing nodes under stable indoor conditions, with observed deviations remaining within the manufacturer-specified uncertainty ranges. Sensor-specific baseline offsets identified during the validation phase were compensated through offset correction during preprocessing.

**Figure 3:**
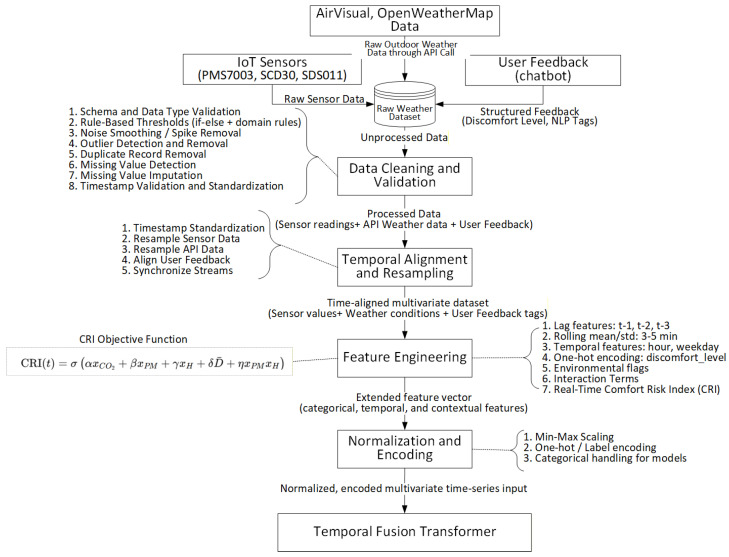
Data -preprocessing pipeline for IoT-based air quality forecasting and context-aware comfort analysis.

The raw data were stored in JSON format and processed through a structured preprocessing pipeline, as illustrated in Figure 3. The preprocessing stage included data cleaning, temporal alignment, noise reduction, and feature engineering. Invalid measurements and outliers were removed, and all heterogeneous data streams were synchronized to a unified 1 min temporal resolution. Contextual and statistical features such as rolling statistics, lag features, and interaction terms were extracted to improve temporal modeling capability. In addition, a composite Comfort Risk Index (CRI) was developed by integrating environmental measurements with occupant discomfort feedback. Finally, normalization and encoding techniques were applied to generate stable inputs for downstream deep learning and fuzzy inference models.

#### 2.2.1. Data Acquisition and Integration

The proposed framework collects real-time environmental and contextual data using a hybrid architecture consisting of IoT sensors, external weather APIs, and occupant feedback. Indoor environmental measurements are acquired using PMS7003 (PM_2.5_, PM_10_) and SCD30 (CO_2_) sensors connected to ESP32 microcontrollers through UART and I^2^C interfaces. Sensor readings are recorded at 5 s intervals and transmitted through MQTT to a centralized IoT gateway. An SDS011 sensor deployed on a separate ESP32 node is used to monitor outdoor particulate matter levels. To incorporate external environmental conditions, AQI, temperature, humidity, wind speed, and atmospheric pressure data are retrieved every 10 min from the public APIs of AirVisual (https://api.airvisual.com/v2/) (accessed on 25 August 2025) and OpenWeatherMap (https://api.openweathermap.org/data/2.5/weather?) (accessed on 25 August 2025). The API responses are received in JSON format and temporally aligned with the indoor sensor streams. A mobile-integrated chatbot is used to collect occupant discomfort feedback under different indoor conditions. Free-text responses (e.g., “The air feels stuffy”) are processed using a lightweight NLP module to generate discomfort labels. All feedback entries are time-stamped and synchronized with the environmental measurements to construct a unified multivariate time-series dataset at 1 min temporal resolution. Sensor nodes were positioned at representative indoor monitoring locations while avoiding direct airflow interference from windows, doors, and HVAC outlets. The data collection process included varying occupancy activities and ventilation conditions to evaluate the robustness of the proposed framework under heterogeneous indoor environments.

The raw dataset consisted of timestamped multivariate environmental observations collected from indoor sensing devices, outdoor environmental APIs, and occupant feedback streams. Indoor measurements included CO_2_, PM_2.5_, PM_10_, humidity, and temperature recorded at 5 s intervals, while outdoor environmental variables including AQI, pressure, wind speed, and ambient weather conditions were retrieved every 10 min through public APIs. Raw measurements occasionally contained missing samples, temporal misalignment, and sensor noise caused by communication delay and environmental fluctuations. Therefore, preprocessing operations including timestamp synchronization, missing-value handling, outlier filtering, normalization, and temporal aggregation were applied to generate a unified 1 min resolution multivariate time-series dataset suitable for forecasting and adaptive control.

#### 2.2.2. Data Cleaning and Validation

The raw dataset was subjected to structured cleaning and validation to ensure integrity and consistency across sensor, API, and user feedback streams. To further improve measurement reliability, baseline-corrected sensor readings were used throughout the analysis. Sensor drift was monitored during the deployment period through periodic cross-comparison of co-located sensing nodes. No substantial baseline drift requiring recalibration was observed during the 30-day deployment period. Potential cross-sensitivity effects caused by humidity and environmental fluctuations were mitigated through temporal filtering, feature normalization, and multivariate data fusion. These procedures reduced the influence of short-term sensor instability before forecasting and control model development.

Domain-specific range filters clip PM_2.5_ to [0, 500] µg/m^3^, CO_2_ to [350, 10,000] ppm, humidity to [0, 100]%, and temperature to [−10, 50] °C. Outliers are further detected using the z-score method (|z|>3). To reduce sensor noise, an α–β filter is applied to CO_2_ and PM readings, and a Savitzky–Golay filter (window = 9, polyorder = 2) smooths high-frequency PM fluctuations.

Duplicates are removed based on timestamp and feature hash. Missing values are interpolated for continuous variables and forward-filled for categorical fields such as discomfort labels. Records outside the deployment window or with unresolved critical fields (e.g., timestamps) are discarded before proceeding to temporal alignment.

#### 2.2.3. Temporal Alignment and Resampling

To align data streams with different sampling rates, all sources are synchronized to a common 1 min temporal resolution. Sensor data from the SCD30, SDS011, and PMS7003 devices initially sampled at 5 s time intervals are resampled and linear interpolation is used to fill the gaps between the windows. To maintain continuity of the API data, the AirVisual and the OpenWeatherMap API data are forward-filled and updated every 10 min. The user feedback (gathered with the help of a chatbot at random times) is mapped to the closest minute and back-filled within a short interval of validity (e.g., 10 min) to indicate the sense of permanent discomfort. All timestamps are reported in Coordinated Universal Time (UTC) and formatted according to the ISO 8601 standard [27]. The aligned streams consisting of sensor data, API context, and user feedback are merged using outer joins on UTC timestamps, producing a synchronized multivariate time-series dataset suitable for downstream modeling. Indoor sensing devices were configured with a 5 s acquisition interval to capture short-term environmental fluctuations associated with occupant movement, pollutant generation events, ventilation changes, and transient indoor activities such as cooking, cleaning, and door/window operation. Although the final forecasting and control framework operated on a 1 min aligned dataset, high-frequency acquisition improved temporal observability during raw data collection and reduced the risk of missing rapid environmental transitions. After acquisition, the raw measurements were temporally aggregated and synchronized into 1 min intervals to reduce communication overhead, memory usage, sensor noise sensitivity, and computational complexity for edge-based forecasting and adaptive fuzzy control deployment. The proposed multirate sensing strategy therefore balances environmental responsiveness during acquisition with lightweight processing requirements within the proposed IoT-edge architecture.

#### 2.2.4. Feature Engineering and CRI

Derived features are generated from the aligned time-series dataset to model temporal patterns and contextual dependencies. Lag features for PM_2.5_, CO_2_, and humidity (i.e., x(t−1) to x(t−3)) are used to capture short-term temporal dependencies. Rolling statistics (mean and standard deviation over 3, 5, and 10 min windows) characterize local variability. Cyclical time features (hour of day and day of week) are encoded using sine and cosine transforms. User discomfort labels are one-hot encoded, while interaction terms such as $xPM\left(t\right)\cdot x_{H}\left(t\right)$ are included to capture compound environmental effects. The CRI is introduced as a composite feature integrating environmental measurements with user perception.

The CRI is designed to quantify real-time indoor discomfort by combining CO_2_, PM_2.5_, humidity, and smoothed user feedback into a single interpretable score. Interaction terms are incorporated to represent dependencies such as humidity-amplified particulate discomfort. The resulting value is passed through a sigmoid transformation to produce a normalized score in the interval [0, 1], where higher values indicate higher discomfort levels. CRI, as defined in Equation (1), is used both as a prediction target and as an input to the fuzzy inference system.

(1) $$\mathrm{CRI}\left(t\right)=\sigma \left(\alpha x_{CO_{2}}\left(t\right)+\beta x_{PM}\left(t\right)+\gamma x_{H}\left(t\right)+\delta \bar{D}\left(t\right)+\eta x_{PM}\left(t\right)x_{H}\left(t\right)\right)$$

where $x_{CO_{2}}\left(t\right)$, $x_{PM}\left(t\right)$, and $x_{H}\left(t\right)$ are min–max normalized inputs for CO_2_, PM_2.5_, and humidity at time *t*, respectively. $\bar{D}\left(t\right)$ represents the exponentially weighted moving average of user discomfort over the previous *N* observations, while α, β, γ, δ, and η denote weighting coefficients associated with each variable. The sigmoid function σ(·) maps the CRI score to the interval [0,1].

The proposed CRI formulation is supported by previous IEQ studies integrating objective environmental measurements and subjective comfort assessment. Specifically, Zhu and Li [28] proposed an IAQ evaluation index linking pollutant indicators with occupant comfort evaluation, while Heinzerling et al. [29] reviewed existing IEQ weighting schemes and concluded that no universally accepted weighting framework currently exists. Following these principles, the proposed CRI incorporates CO_2_, PM_2.5_, humidity, temperature, and occupant discomfort feedback. The relative importance assigned to these variables was guided by findings reported in the literature, which consistently identify CO_2_ and PM_2.5_ as major determinants of perceived indoor air quality and health risk, while temperature and humidity primarily influence thermal comfort and occupant satisfaction. Occupant discomfort feedback was explicitly included to preserve the human-centric nature of the proposed framework. The final coefficients adopted in this study were therefore adapted from the weighting philosophies reported in previous IEQ studies [28,29] and subsequently refined through expert-guided sensitivity analysis to ensure balanced CRI dynamics and stable control responses across heterogeneous indoor environments.

The coefficients satisfied α+β+γ+δ+η=1, and the same set of coefficients was retained throughout all experiments to ensure consistency during forecasting and control evaluation.

The user discomfort score D(t) is obtained by mapping text-based feedback (e.g., “The air feels stuffy”) to discrete levels: None = 0, Moderate = 0.5, and High = 1. These scores are smoothed using exponential decay, as defined in Equation (2).

(2) $$\bar{D}\left(t\right)=\frac{1}{Z}\sum_{i=1}^{N}\lambda^{i}\cdot D\left(t-i\right),\;Z=\sum_{i=1}^{N}\lambda^{i},\;\lambda \in \left(0,1\right]$$

Algorithm 1 outlines the procedure used to compute the CRI from environmental and contextual inputs.

**Algorithm 1** CRI computation at time *t***Require:**  1:Normalized inputs: $x_{CO_{2}}\left(t\right)$, $x_{PM}\left(t\right)$, $x_{H}\left(t\right)$ 2:Discomfort history: D(t−1),…,D(t−N) 3:Hyperparameters: λ∈(0,1] and weights θ=α,β,γ,δ,η**Ensure:** CRI(t)∈[0,1]  4:Compute exponentially weighted discomfort score:$$\bar{D}\left(t\right)=\frac{1}{Z}\sum_{i=1}^{N}\lambda^{i}\cdot D\left(t-i\right)$$ 5:Compute interaction term:$$I\left(t\right)=x_{PM}\left(t\right)\cdot x_{H}\left(t\right)$$ 6:Compute weighted risk score:$$z\left(t\right)=\alpha x_{CO_{2}}\left(t\right)+\beta x_{PM}\left(t\right)+\gamma x_{H}\left(t\right)+\delta \bar{D}\left(t\right)+\eta I\left(t\right)$$ 7:Apply sigmoid activation:$$\mathrm{CRI}\left(t\right)=\frac{1}{1+e^{-z(t)}}$$  **return** CRI(t)

The proposed CRI was empirically validated against both subjective occupant discomfort and objective IAQ guideline indicators using the deployment dataset. CRI values were compared across chatbot-derived discomfort categories (None, Moderate, and High), with higher discomfort levels consistently associated with higher CRI values. In addition, CRI behaviour was evaluated under IAQ exceedance conditions, including CO_2_ concentrations above 1000 ppm and PM_2.5_ concentrations exceeding the WHO 24 h guideline value of 15 μg/m^3^. Spearman’s rank correlation and Kruskal–Wallis analyses confirmed statistically significant associations between CRI, occupant discomfort, and deteriorating IAQ conditions. As summarized in Table 2, the proposed findings support the validity of CRI as a practical comfort-aware indicator for adaptive IAQ optimization.

#### 2.2.5. Normalization and Encoding

To self-scale the input and to make the calculations numerically stable, all continuous variables, such as CO_2_, PM_2.5_, humidity, temperature, and derived statistics, are rescaled to a range between [0, 1], as with min–max normalization. One-hot encoding is used on the categorical variables, e.g., user discomfort levels. Meanwhile, any attribute of time (e.g., hour of day, day of week) is coded in sine and cosine to maintain cyclical continuity. The false informational items like a flag of a Boolean type, interaction terms, composite measures like CRI are left as-is or normalized where complimentary. The standardization allows for efficient training and inference of models both in the edge and cloud platform.

### 2.3. Temporal-Aware Predictive Modeling

The predictive framework for multivariate forecasting of IAQ indicators and occupant discomfort is designed to capture temporal dependencies among environmental variables, user feedback, and CRI, while remaining suitable for deployment on resource-constrained edge devices. The framework operates in three stages: a cloud-trained TFT captures multistep temporal dependencies, knowledge distillation produces a lightweight Bi-LSTM model for edge inference, and a fuzzy inference system translates predictions into context-aware actuation. The resulting CRI and environmental forecasts guide the fuzzy controller in making real-time decisions for ventilation, filtration, and other interventions.

#### 2.3.1. Cloud-Based Training of the Temporal Fusion Transformer

A TFT is trained on the preprocessed dataset to forecast multivariate environmental states and the CRI. TFT is selected for its ability to capture both long-term dependencies and short-term dynamics via gated residual networks, variable selection networks, and attention mechanisms. It also enables interpretable forecasting by assigning learned importance weights to input variables.

Let $X_{1:T}={\left\{x\left(t\right)\right\}}_{t=1}^{T}$ denote the input sequence over *T* time steps, where each $x\left(t\right)\in R^{d}$ is a *d*-dimensional feature vector. The model predicts a future target sequence ${\hat{Y}}_{T+1:T+H}$ over the prediction horizon *H* as follows:

$${\hat{Y}}_{T+1:T+H}={\mathrm{TFT}}_{\theta }\left(X_{1:T},s_{T+1:T+H}\right)$$

where $s_{T+1:T+H}$ includes known future covariates (e.g., time encodings) and θ denotes the model parameters. The training is performed via backpropagation through time, minimizing the multistep mean squared error (MSE) loss:

$$L_{\mathrm{MSE}}=\frac{1}{H}\sum_{h=1}^{H}{∥{\hat{y}}_{T+h}-y_{T+h}∥}^{2}$$

To optimize the hyperparameters, a grid Bayesian search and regularization techniques have been applied to reduce overfitting. Although TFT offers high accuracy and interpretability, its computational complexity renders it unsuitable for edge deployment. To this end, we aim to motivate the use of knowledge distillation in the next stage.

#### 2.3.2. Knowledge Distillation to Edge-Deployed Model

To enable real-time inference on edge devices, the cloud-trained TFT is compressed using a knowledge distillation framework. This transfers the predictive behavior of the high-capacity teacher model into a lightweight student model suitable for platforms such as ESP32 and Raspberry Pi. The student model receives the same multivariate input sequence $X_{1:T}={\left\{x\left(t\right)\right\}}_{t=1}^{T}$, which includes normalized sensor data, engineered features, and the CRI. The distillation target is the teacher’s output ${\hat{Y}}^{\left(T\right)}=\left\{{\hat{y}}_{T+1}^{\left(T\right)},\ldots ,{\hat{y}}_{T+H}^{\left(T\right)}\right\}$ over the prediction horizon *H*. A lightweight Bi-LSTM student model $Student_{\phi }\left(\cdot \right)$ is trained using Equation (3) to approximate the teacher’s predictions by minimizing the distillation loss.

(3) $$\begin{array}{cc} {\hat{Y}}^{\left(S\right)} & ={\mathrm{Student}}_{\phi }\left(X_{1:T}\right)\\ L_{\mathrm{KD}} & =\frac{1}{H}\sum_{h=1}^{H}{∥{\hat{y}}_{T+h}^{\left(S\right)}-{\hat{y}}_{T+h}^{\left(T\right)}∥}^{2} \end{array}$$

Once trained, the student model achieves near-TFT performance with significantly reduced memory and compute overhead, enabling continuous edge-based forecasting of CRI and pollutant levels for real-time fuzzy control.

### 2.4. Proposed Fuzzy Logic-Based Control and Actuation Model

The final stage of the proposed framework employs a fuzzy logic controller to convert predicted environmental indicators into real-time control actions. The controller operates on the outputs of the edge-deployed student model, namely CRI, CO_2_, PM_2.5_, humidity, and temperature. Triangular membership functions $\mu_{A_{j}}\left(x\right)$ are used to fuzzify crisp inputs into linguistic categories such as Low, Moderate, and High. For example, CRI is mapped to Low/Moderate/High, CO_2_ to Normal/Elevated/Unhealthy, and temperature to Cold/Moderate/Hot. Each fuzzy set $A_{j}$ is defined as follows:

$$\mu_{A_{j}}\left(x\right)=\left\{\begin{array}{cc} 0, & x\leq a\;\mathrm{or}\;x\geq c\\ \frac{x-a}{b-a}, & a<x\leq b\\ \frac{c-x}{c-b}, & b<x<c \end{array}\right.$$

The fuzzy membership ranges and inference rules were designed using established indoor air quality thresholds, thermal comfort recommendations, and expert-driven heuristic control knowledge obtained from prior fuzzy HVAC and IAQ control studies. Pollutant-related membership boundaries were aligned with commonly adopted indoor exposure and comfort ranges for CO_2_, PM_2.5_, humidity, and temperature. The triangular membership functions were iteratively refined during experimental evaluation to ensure stable actuator transitions, reduced oscillatory behavior, and consistent control sensitivity under dynamic indoor conditions.

Mamdani-type fuzzy inference rules are evaluated using:

$$\omega_{r}=\min\left(\mu_{A_{1r}}\left(x_{1}\right),\ldots ,\mu_{A_{nr}}\left(x_{n}\right)\right)$$

Fuzzy outputs are aggregated using max–min composition:

$$\mu_{B}\left(y\right)=\max_{r=1}^{R}\min\left(\omega_{r},\mu_{B_{r}}\left(y\right)\right)$$

The aggregated output was defuzzified using the centroid method:

$$y^{*}=\frac{\int y\mu_{B}\left(y\right)\;dy}{\int \mu_{B}\left(y\right)\;dy}$$

The resulting $y^{*}$ values are mapped to real-time actuator commands. Table 3 summarizes the output control variables and their corresponding states.

The generated control commands are dispatched to IoT-enabled devices such as fan controllers, air purifiers, and smart HVAC systems. The fuzzy logic framework enables adaptive and interpretable control without requiring static threshold-based operation.

Table 4 summarizes the fuzzy input/output variables and their linguistic partitions.

The fuzzy inference engine transforms forecasted IAQ indicators into real-time actuator commands, as outlined in Algorithm 2. The controller operates on predicted CRI, CO_2_, PM_2.5_, humidity, and temperature values represented using triangular membership functions.
**Algorithm 2** Fuzzy inference engine for indoor air quality controlPredicted inputs: CRI, CO_2_, PM_2.5_, Humidity, Temperature Crisp actuator outputs: $y_{\mathrm{fan}},\;y_{\mathrm{purifier}},\;y_{\mathrm{vent}},\;y_{\mathrm{hvac}}$ **Sample fuzzy rules:** The rule base was constructed to prioritize occupant comfort stabilization while minimizing unnecessary actuator operation under dynamically changing indoor conditions.
R_1_: if CRI is high and CO_2_ is unhealthy and PM_2.5_ is poor, then fan = high and purifier = highR_2_: if CRI is moderate and CO_2_ is elevated, then fan = medium and purifier = mediumR_3_: if CRI is low and CO_2_ is normal, then fan = low and purifier = OffR_4_: if temperature is hot, then HVAC = coolR_5_: if humidity is humid and CO_2_ is unhealthy, then ventilation = full


**Figure 4:**
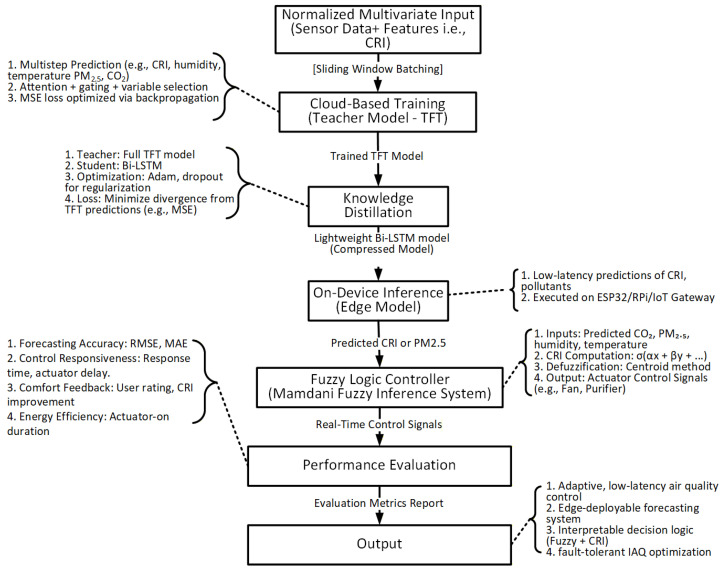
End-to-end predictive analytics pipeline for intelligent indoor air quality management.

Figure 4 illustrates the complete pipeline including TFT training, knowledge distillation, edge-level inference, CRI-guided fuzzy control, and real-time actuation.

In practical operation, the proposed optimization framework continuously predicts short-term indoor environmental conditions and dynamically adjusts actuator behavior before severe comfort degradation occurs. The predicted CRI, CO_2_, PM_2.5_, humidity, and temperature values are evaluated by the Mamdani fuzzy inference engine to determine appropriate ventilation, purification, and HVAC control actions. For example, elevated predicted CO_2_ and CRI levels trigger increased ventilation and fan intensity, while high PM_2.5_ concentrations activate purifier operation with adaptive intensity control. Unlike conventional threshold-based systems that respond only after pollutant limits are exceeded, the proposed predictive optimization framework performs proactive and gradual actuator modulation based on evolving environmental trends. Experimental evaluation demonstrates that the optimized fuzzy control strategy produces smoother actuator transitions, lower CRI variability, improved comfort regulation, and reduced unnecessary actuator operation compared to the baseline threshold-based approach. The above results indicate that the integration of predictive forecasting, CRI-guided reasoning, and adaptive fuzzy actuation enables more stable and energy-aware IAQ management under dynamic indoor conditions.

## 3. Development Environment

The proposed hybrid framework was implemented using a distributed architecture that combines cloud-based training with edge-level inference and control. The core predictive model was developed in Python 3.10 using the PyTorch Lightning library. A TFT was trained on Google Colab with an NVIDIA A100 GPU, and the distilled Bi-LSTM student model was exported for edge deployment.

Edge operations were performed on a Raspberry Pi 4B (8 GB RAM, Ubuntu 22.04), which handled real-time data ingestion, prediction, and actuation. Indoor air quality was monitored via ESP32-WROOM nodes connected to PMS7003 (PM_2.5_/PM_10_), SCD30 (CO_2_), and DHT22 (temperature/humidity) sensors over UART/I^2^C. External PM data were collected using an SDS011 sensor on a separate ESP32 node.

Environmental context (e.g., AQI, weather conditions) was fetched from OpenWeatherMap and AirVisual APIs every 10 min using HTTP GET. All data were transmitted via MQTT and aggregated at 1 min resolution on the gateway device. The fuzzy logic controller was implemented using the Scikit-Fuzzy library, employing Mamdani inference and triangular membership functions. Fan speed, purifier intensity, HVAC mode, and ventilation level were all mapped to discrete actuator commands and sent to smart Wi-Fi-enabled IoT relays. InfluxDB and grafana dashboards offered real-time monitoring and logging. Table 5 represents a summary of hardware requirements and software requirements.

### Dataset Summary

The dataset was collected over a continuous 30-day deployment period using the proposed IoT-based indoor air-quality-monitoring framework under varying indoor occupancy and ventilation conditions. The dataset consists of multivariate time-series observations sampled at a 1 min temporal resolution, resulting in a total of 43,200 synchronized records. The collected data include indoor environmental measurements such as CO_2_, PM_2.5_, PM_10_, humidity, and temperature acquired through IoT sensor nodes. DHT22 sensors were used for temperature and humidity acquisition. In addition, outdoor contextual information including AQI, atmospheric pressure, temperature, and wind speed was retrieved using public weather APIs. Occupant discomfort feedback collected through the chatbot interface was also incorporated and temporally smoothed to preserve contextual continuity. Derived features such as the CRI were computed and included for downstream forecasting and fuzzy control tasks. All records were time-stamped in UTC and stored in JSON format to ensure compatibility and interoperability across cloud and edge deployment environments. A detailed overview of the dataset and sensing infrastructure is presented in Table 6 and Table 7.

## 4. Results and Discussion

This section presents a comprehensive evaluation of the proposed fuzzy hybrid control architecture for real-time IAQ optimization. The system is evaluated across multiple performance dimensions, including forecasting accuracy, control latency, energy-aware actuation, rule interpretability, and resilience under dynamic indoor conditions. Quantitative metrics such as CRI, MAE, actuator energy usage, and system response time are compared against baseline predictive and control approaches. Additional experiments are conducted to evaluate the contribution of optimization modules, edge deployment capability, and fallback control strategies. The scalability and resilience of the framework on resource-constrained edge platforms are further validated through real-time profiling and cross-environment evaluation.

### 4.1. Control Performance Evaluation

The proposed fuzzy control framework was evaluated through comparative experiments against a conventional threshold-based baseline controller operating under identical environmental input streams and deployment conditions. Both controllers received the same synchronized indoor sensor measurements, outdoor environmental context, and occupant feedback inputs throughout the evaluation period to ensure consistent comparison conditions.

**Figure 5:**
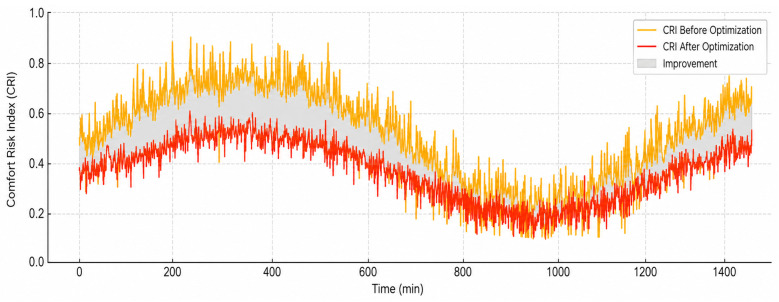
Temporal comparison of the CRI before and after optimization using fuzzy control.

The baseline strategy utilized fixed threshold-triggered actuator control based on predefined pollutant limits and comfort ranges, whereas the proposed framework employed predictive CRI-driven fuzzy reasoning with adaptive actuator modulation. The evaluation focused primarily on relative comfort stabilization, actuator operating behavior, and real-time control responsiveness. Figure 5 illustrates the effect of the fuzzy controller on the CRI. Before actuation, the CRI exhibits significant temporal fluctuations with strong peaks during pollutant events. After the integration of fuzzy control, the CRI profile becomes smoother with reduced peak intensity. The fuzzy control framework significantly reduced CRI fluctuations and improved comfort stabilization under varying indoor environmental conditions. Furthermore, the CRI validation analysis demonstrated that higher CRI values were significantly associated with increased occupant discomfort and periods exceeding recommended IAQ thresholds. These findings support the practical relevance of the proposed CRI as a comfort-aware control indicator for adaptive indoor environmental management.

**Figure 6:**
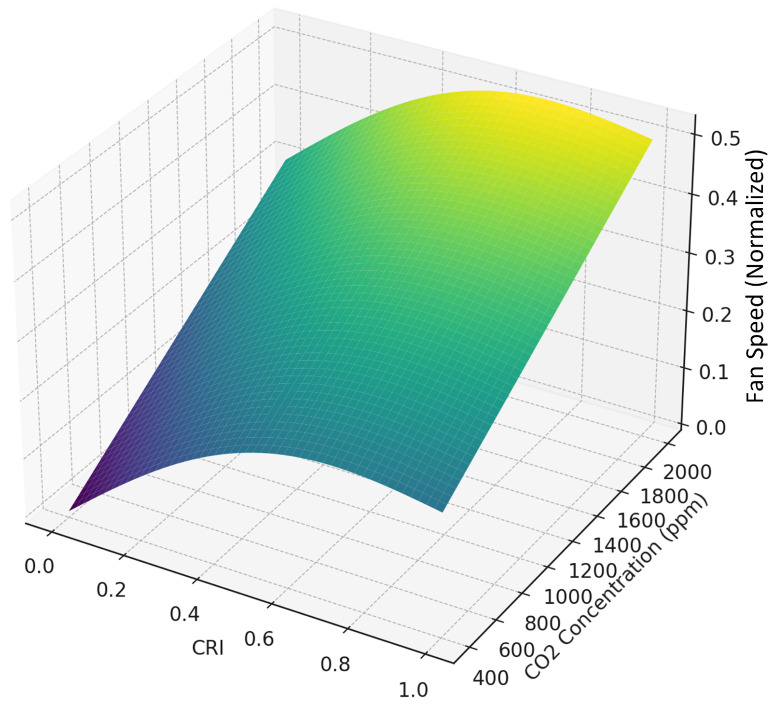
Fan speed output as a function of CRI and CO_2_ concentration (ppm). The color gradient represents the normalized fan speed predicted by the fuzzy inference system, with darker colors indicating lower output values and brighter colors indicating higher output values.

Figure 6 presents the fuzzy inference surface for fan speed as a function of CRI and CO_2_ concentration. The output exhibits a nonlinear increase in control intensity under worsening environmental conditions. The surface is generated using Mamdani inference with triangular membership functions and centroid defuzzification, enabling smooth actuator transitions and interpretable rule-to-output mapping.

Table 8 summarizes the comparative performance of the proposed deep–fuzzy hybrid system under baseline and fuzzy control strategies. Forecasting accuracy improves, with MAE decreasing from 0.115 to 0.078 and RMSE decreasing from 0.182 to 0.121. MAPE is also reduced from 9.40% to 5.20%, indicating improved prediction stability.

Control-related metrics also improve, with CRRR increasing from 0.02 to 0.27 and CPR increasing from 0.68 to 0.91, indicating more stable comfort maintenance during the deployment period. The optimized fuzzy control strategy also reduced estimated actuator energy usage by lowering unnecessary switching events and actuator operating intensity. This behavior is reflected by the reduction in the estimated actuator energy intensity (EEI) metric from 0.84 kWh to 0.39 kWh during the evaluation period, indicating lower relative actuator operating demand under adaptive fuzzy control.

To evaluate the effect of preprocessing operations on forecasting performance, an ablation analysis was conducted using three dataset configurations: raw sensor measurements, partially filtered data, and fully processed temporally aligned data. The partially filtered dataset included outlier removal and missing-value interpolation, while the fully processed dataset additionally incorporated Savitzky–Golay smoothing, temporal synchronization, and 1 min aggregation. The results indicate that preprocessing improved temporal stability and reduced high-frequency sensing noise commonly associated with low-cost environmental sensors. Consequently, forecasting performance improved progressively across the preprocessing stages. In addition, the 1 min temporal aggregation reduced communication overhead, memory usage, and computational complexity for edge deployment while preserving the dominant environmental dynamics required for adaptive IAQ forecasting and control. The comparative forecasting performance across different preprocessing configurations is summarized in Table 9.

It should be noted that the preprocessing pipeline contributed substantially to the overall forecasting performance. As presented in Table 9, the inclusion of data cleaning, noise filtering, outlier handling, interpolation, and temporal aggregation reduced prediction error significantly compared with the raw-data configuration (MAE reduced from 0.104 to 0.078). This improvement is partly attributable to variance reduction and noise suppression in the low-cost sensor measurements, which simplifies the forecasting task by providing a more stable representation of the underlying environmental dynamics. Therefore, the reported forecasting accuracy reflects the combined contribution of both the preprocessing framework and the proposed predictive architecture rather than the predictive model alone.

### 4.2. Sensor-Driven CRI Response Analysis

**Figure 7:**
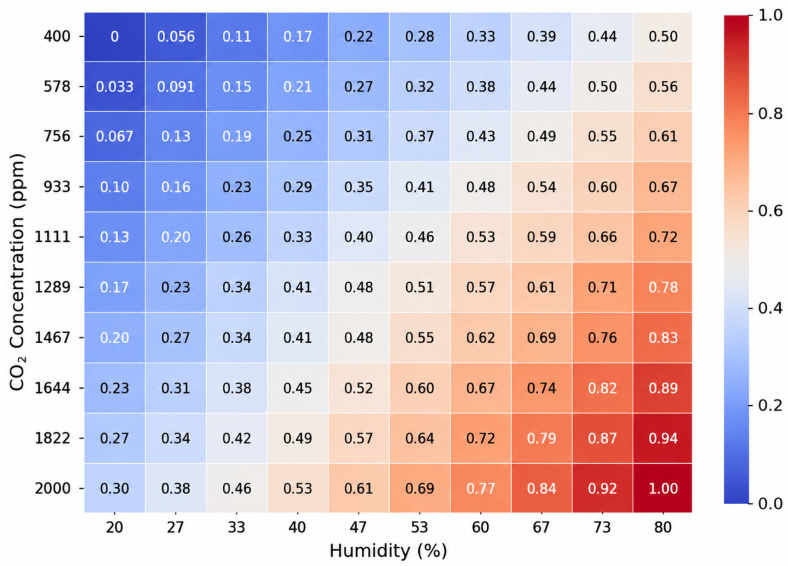
Heatmap showing CRI variation with respect to CO_2_ (ppm) and relative humidity (%).

Figure 7 illustrates the variation in CRI with respect to CO_2_ concentration and relative humidity. The CRI exhibits nonlinear behavior, with elevated discomfort regions appearing at CO_2_ levels above 1600 ppm and humidity levels exceeding 70%.

Interaction terms such as $x_{{\mathrm{CO}}_{2}}\cdot x_{H}$ are incorporated to model compound environmental effects within the fuzzy control framework. Triangular membership functions and centroid defuzzification generate smooth control gradients, enabling proportional and context-aware actuator responses under varying environmental conditions.

**Figure 8:**
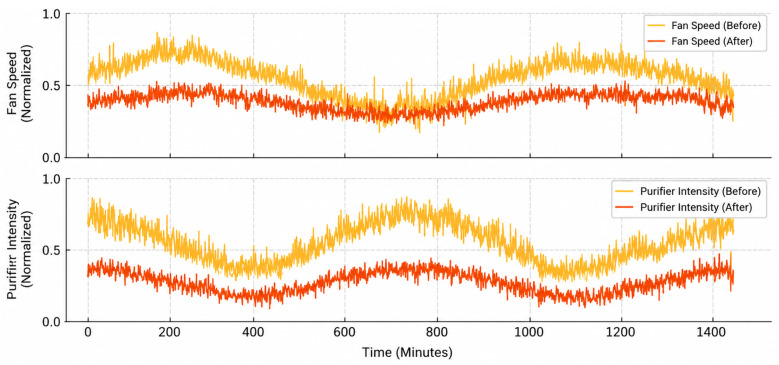
Time-series comparison of actuator intensities for fan speed (**top**) and purifier operation (**bottom**) over a 24 h deployment period.

Figure 8 presents the normalized 24 h operational profiles of fan speed and purifier intensity under baseline and optimized control conditions. The baseline controller exhibits high-amplitude fluctuations and extended peak operation associated with static threshold-based control. In contrast, the optimized fuzzy controller produces smoother actuator transitions and lower duty cycles using predicted discomfort indicators, including CRI, CO_2_, PM_2.5_, humidity, and temperature. This behavior reduces unnecessary actuator operation and supports energy-aware control.

**Figure 9:**
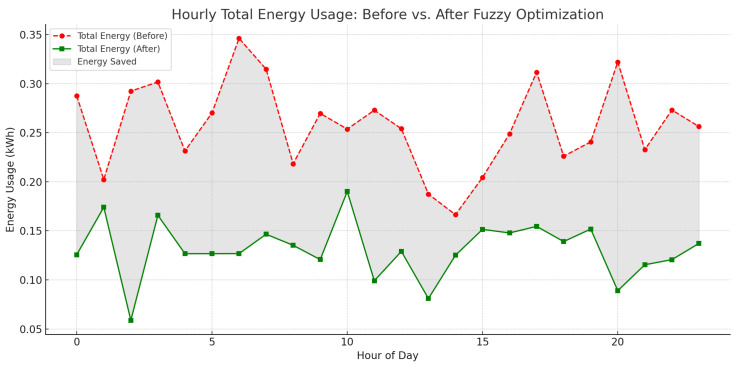
Hourly actuator energy usage before and after fuzzy optimization. Shaded region denotes estimated relative reduction in actuator energy usage.

Figure 9 compares the hourly actuator energy usage before and after fuzzy optimization. The baseline profile exhibits higher variance and peak activity, reflecting reactive and redundant control behavior. The optimized profile is smoother and lower in magnitude, indicating reduced actuator operating intensity under adaptive fuzzy control. The shaded region represents the estimated relative reduction in actuator energy usage achieved through energy-aware actuation.

### 4.3. Interpretable Fuzzy Reasoning Dynamics

**Figure 10:**
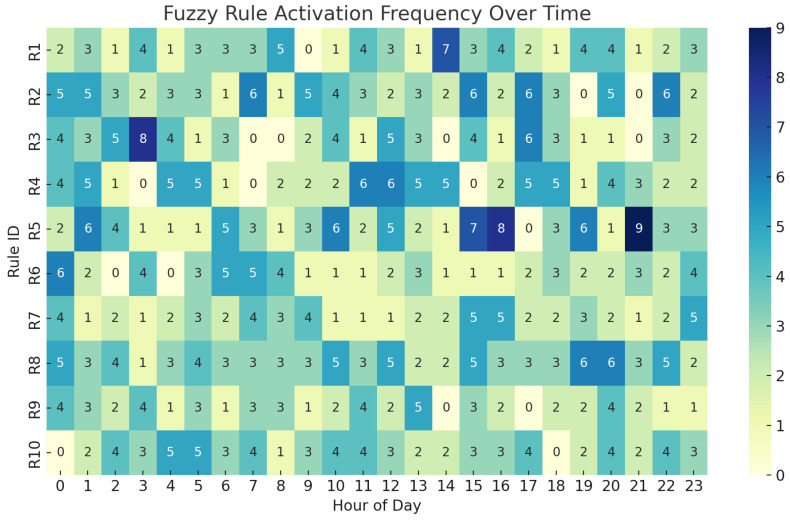
Heatmap of fuzzy rule activation frequency over a 24 h operational window.

Figure 10 illustrates the activation frequency of the fuzzy control rules over a 24 h operational period. The heatmap shows temporal variations in rule activation, reflecting the ability of the fuzzy controller to adapt to changing environmental conditions and occupant comfort requirements. Increased activation of specific rules during high-risk periods demonstrates the dynamic responsiveness of the fuzzy inference engine under varying IAQ conditions. The observed rule activation patterns further validate the consistency and practical behavior of the expert-defined fuzzy rule base under varying environmental conditions.

**Figure 11:**
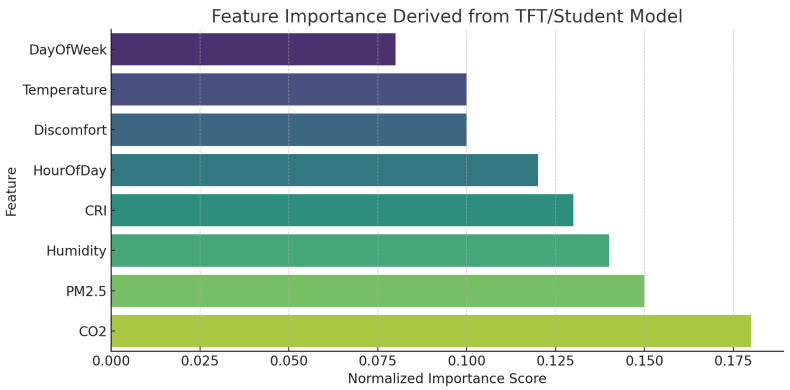
Normalized feature importance scores from the TFT and student models. CO_2_, PM_2.5_, and CRI are dominant predictors, validating alignment with discomfort-driven control.

Figure 11 presents the normalized feature importance scores obtained from the Temporal Fusion Transformer (TFT) and student models. CO_2_, PM_2.5_, and CRI exhibit the highest contribution to forecasting performance, indicating the dominant influence of pollutant concentration and discomfort indicators within the predictive framework. This observation is consistent with the input selection strategy used in the fuzzy controller, demonstrating alignment between the forecasting and control layers.

### 4.4. Energy-Aware Intervention Analysis

**Figure 12:**
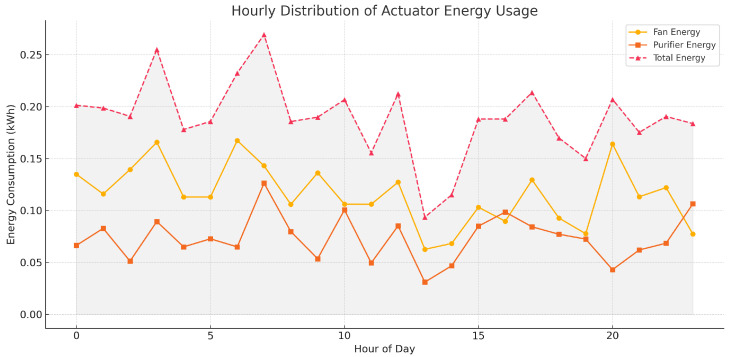
Hourly energy usage distribution for fan and purifier actuators.

Figure 12 presents the hourly energy usage distribution of fan and purifier actuators over a 24 h operational period. Variations in actuator usage follow the temporal occupancy and pollutant patterns observed within the monitored environment. Higher actuator activity is generally associated with elevated CRI and CO_2_ levels. These trends highlight the importance of temporally adaptive control for balancing occupant comfort and energy-aware operation.

Table 10 compares three IAQ intervention strategies, namely HVAC operation, natural ventilation, and fan boosting, across five indoor zones. A relative efficiency metric defined as ΔCRI/ΔEnergy is used to represent the ratio of comfort improvement to estimated additional actuator energy usage.

Natural ventilation demonstrates higher relative efficiency under lower occupancy conditions, particularly in the office and bedroom environments. Fan boosting provides moderate short-term improvement with lower operational intensity, while HVAC-based intervention achieves larger CRI reduction at a comparatively higher estimated energy cost. These observations indicate that different intervention strategies may be preferable under different occupancy and environmental conditions.

### 4.5. Fault Tolerance and System Resilience

**Figure 13:**
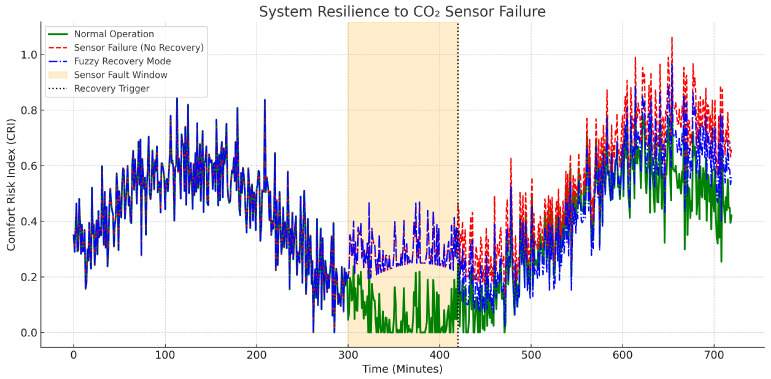
Resilience visualization of the fuzzy hybrid system under CO_2_ sensor failure.

Figure 13 illustrates the system response during a simulated CO_2_ sensor failure over a 12 h period (720 min). The experiment consists of three operating phases: normal operation, unmanaged sensor failure, and recovery using fuzzy fallback control. Under normal conditions, the CRI remains relatively stable. At minute 300, a simulated sensor dropout causes a rapid increase in CRI due to degraded environmental observability. At minute 420, the fallback control mechanism is activated using redundant inputs and fuzzy inference, resulting in gradual CRI recovery. The shaded region represents the fault exposure interval between minutes 300 and 420. These observations demonstrate the resilience of the proposed framework under incomplete sensing conditions.

**Figure 14:**
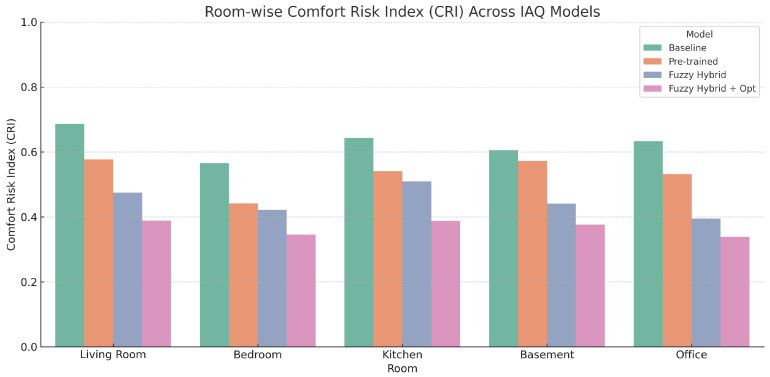
Comparison of CRI across five indoor zones under four model configurations: rule-based, deep model, fuzzy hybrid, and optimized fuzzy hybrid. Lower values indicate better comfort regulation.

Figure 14 compares CRI values across five indoor environments, namely the living room, bedroom, kitchen, basement, and office, using four model configurations: rule-based baseline, pretrained deep model, fuzzy hybrid (without optimization), and optimized fuzzy hybrid.

The rule-based baseline maintains acceptable CRI levels but lacks adaptive multivariate control capability. The pretrained deep model improves forecasting performance but does not provide intelligent actuation. The fuzzy hybrid framework reduces CRI through interpretable rule-based control, while the optimized fuzzy hybrid configuration consistently achieves lower CRI values, particularly in high-risk environments such as the kitchen and basement. These results demonstrate the ability of the proposed framework to regulate IAQ under varying indoor conditions.

**Figure 15:**
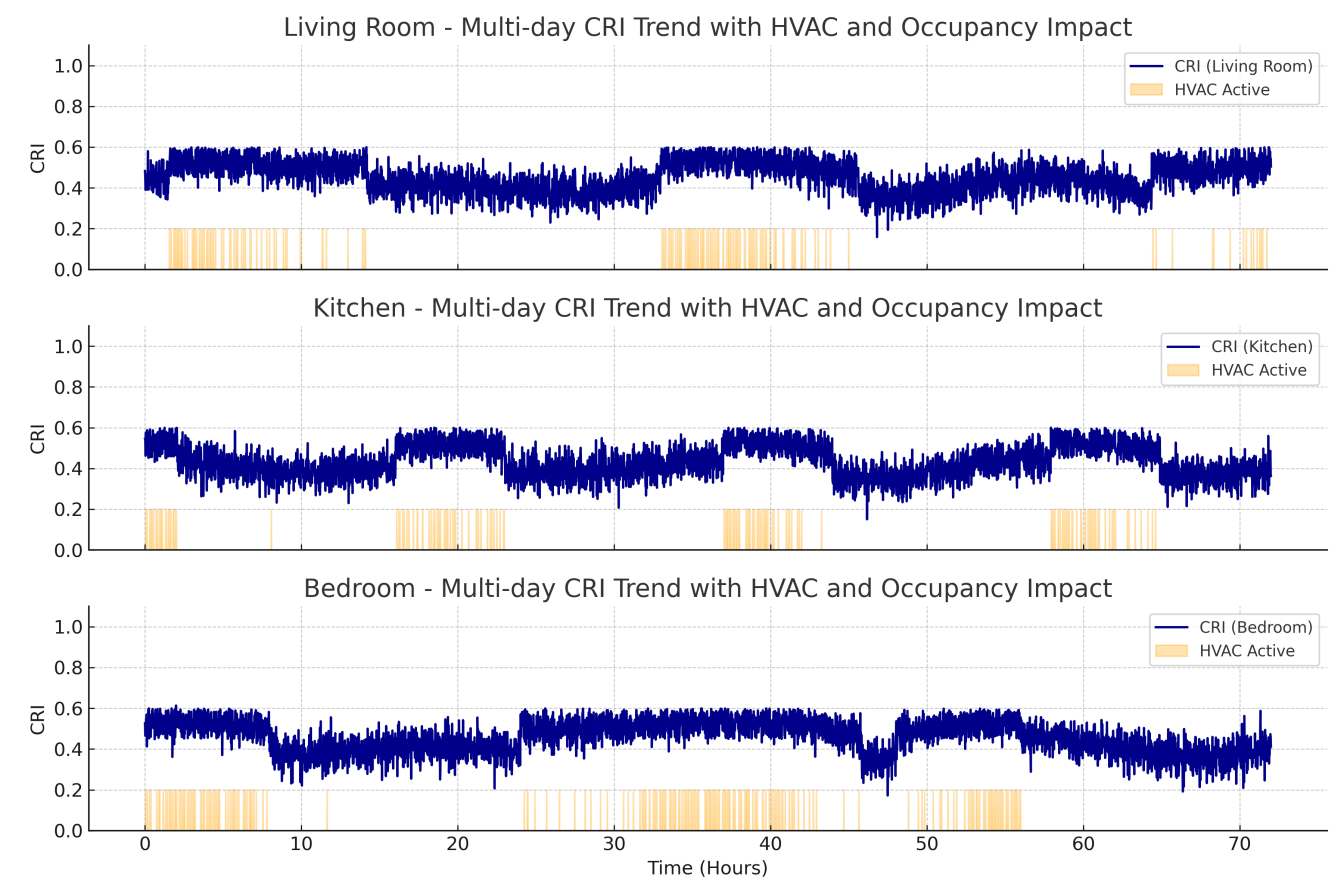
72 h CRI simulation for three rooms under dynamic occupancy.

Figure 15 presents a 72 h CRI simulation for the living room, kitchen, and bedroom under dynamic occupancy conditions. Variations in CRI are influenced by pollutant fluctuations and room usage patterns. The shaded regions indicate periods of HVAC operation when CRI exceeds 0.6, resulting in observable reduction in elevated discomfort periods.

Distinct room-specific behavior is observed across the monitored environments. The kitchen exhibits rapid pollutant-driven CRI spikes, the bedroom demonstrates longer nighttime fluctuations, and the living room shows moderate cyclic variation associated with occupancy activity. These observations highlight the importance of adaptive real-time IAQ control under dynamic environmental conditions.

**Figure 16:**
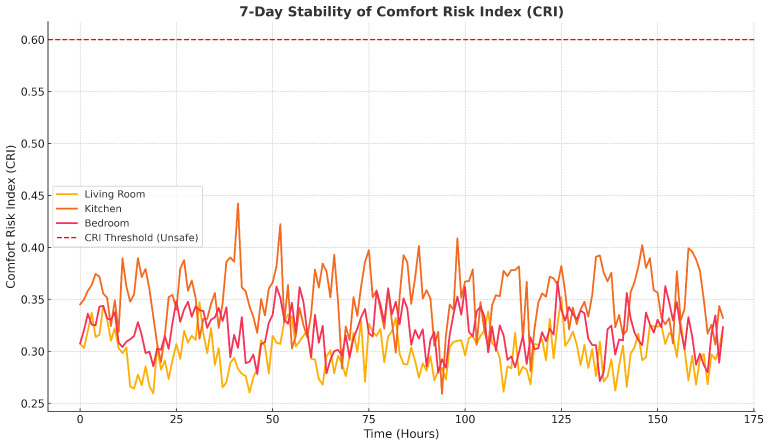
7-day stability of CRI across three indoor zones.

Figure 16 presents a 7-day CRI stability analysis across the living room, kitchen, and bedroom environments. Despite temporal variations in occupancy and pollutant levels, CRI remains below the critical threshold of 0.6 for most operational periods, indicating stable comfort regulation under dynamic indoor conditions.

**Figure 17:**
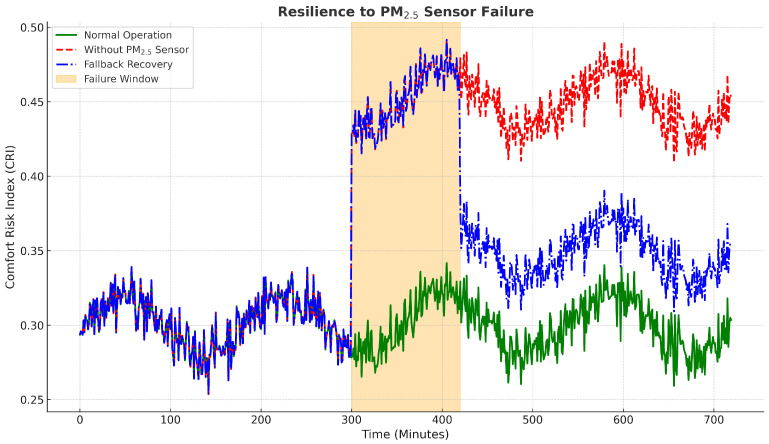
Resilience to PM_2.5_ sensor failure over a 12 h simulation.

Figure 17 demonstrates the system response during a simulated PM_2.5_ sensor failure. A CRI increase occurs after sensor loss at minute 300, while recovery begins at minute 420 using fallback fuzzy control logic. This behavior demonstrates the robustness of the proposed framework under multi-sensor disruption scenarios.

### 4.6. Multi-Objective System Evaluation

Table 11 compares the proposed fuzzy-hybrid-enabled optimization model with baseline statistical, deep learning, and ensemble approaches across multiple performance metrics. While deep learning models provide competitive forecasting accuracy, they generally exhibit higher latency, larger model size, and lower interpretability. The proposed framework achieves low MAE (0.060), reduced inference latency (295 ms), lower estimated actuator energy usage (0.39 kWh/day), interpretability, and resilience to sensor failure, demonstrating its suitability for real-time edge IAQ control.

**Figure 18:**
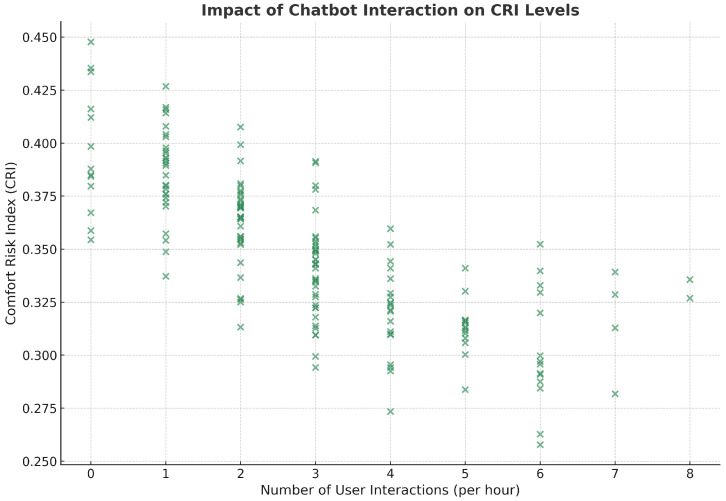
Relationship between chatbot engagement and CRI. Increased interaction is generally associated with lower CRI levels, supporting the framework’s human-in-the-loop adaptability.

Figure 18 illustrates the relationship between chatbot interaction frequency and CRI. Increased user interaction is generally associated with lower CRI levels, indicating that occupant feedback contributes to adaptive control behavior within the proposed framework. This observation supports the human-centric design of the system, where user engagement is incorporated into the IAQ decision-making process.

Although the proposed framework demonstrated improved comfort stabilization and reduced estimated actuator operating intensity, future work should incorporate direct HVAC power metering, occupancy-normalized benchmarking, and statistically controlled validation experiments across larger deployment environments to further quantify long-term energy and comfort benefits.

## 5. Conclusions

This paper proposed a human-centric deep–fuzzy hybrid framework for real-time IAQ prediction and adaptive control in smart indoor environments. The proposed framework integrates IoT-based environmental sensing, multivariate time-series forecasting, knowledge distillation, and interpretable fuzzy logic control within a unified edge-enabled IAQ management architecture. A teacher–student learning strategy was employed, where a TFT model was used as the teacher network and a lightweight Bi-LSTM model was deployed as the student model for low-latency edge inference. A composite CRI was additionally introduced to combine environmental parameters and occupant discomfort feedback into a unified adaptive control indicator for intelligent IAQ regulation. The experimental results demonstrated forecasting accuracies of 96.3% for CO_2_ and 95.7% for PM_2.5_ prediction while reducing inference latency from 575 ms to 295 ms through knowledge distillation and edge deployment. The major findings and contributions of this work are summarized as follows:

- A unified IoT-edge enabled IAQ optimization framework was developed by integrating multivariate forecasting, knowledge distillation, and Mamdani fuzzy control while enabling low-latency edge deployment through a lightweight Bi-LSTM student model.
- The proposed CRI framework effectively combined environmental measurements and occupant discomfort feedback into a unified comfort-aware control indicator for adaptive IAQ management.
- The fuzzy inference system enabled interpretable and context-aware actuator control for ventilation, purification, and HVAC operation using forecasted environmental conditions and adaptive rule-based reasoning.
- Comparative evaluation against baseline threshold-based control strategies demonstrated smoother actuator transitions, improved comfort stability, reduced CRI fluctuations, and lowered estimated actuator operating intensity during deployment.
- The proposed framework additionally demonstrated resilience under simulated sensor-failure conditions through fallback fuzzy reasoning and adaptive control continuity.

Despite the promising results, several limitations remain. Experimental deployment was conducted within a limited smart-building testbed and may not fully represent large-scale commercial, educational, or industrial environments. In addition, the fuzzy membership functions and rule base were developed using expert-guided heuristic tuning rather than adaptive self-learning optimization. Although practical calibration, baseline correction, and cross-comparison procedures were implemented prior to deployment, the sensing platform relied on low-cost commercial sensors rather than laboratory-grade reference instruments. Consequently, further validation under long-term deployments, diverse occupancy conditions, and reference-grade sensing infrastructure is required. Overall, the proposed framework provides an interpretable, scalable, and energy-aware solution for intelligent IAQ optimization in resource-constrained smart environments. Future work will focus on large-scale real-world deployment, occupancy-aware adaptive control, reinforcement-learning-assisted optimization, and the integration of additional environmental and behavioral sensing modalities for personalized IAQ management.

## Figures and Tables

**Table 1:** Comparative analysis of existing methods and the proposed model.

Paper (Bibitem ID)	Year	Main Focus	AI Techniques	IoT/Edge Deployment	Human-Centric Design	Real-Time Capable	Multi-Objective Optimization	Fault Tolerance	Interpretable Model	Edge Deployable	Robust to Sensor Failure
[12]	2021	IAQ estimation and forecasting	MLP, XGBR, LSTM-wf	Partial	✗	✓	✗	✗	✗	✗	✗
[13]	2019	IAQ forecasting (CO_2_)	ML models	Partial	✗	✓	✗	✗	✗	✗	✗
[14]	2019	Review of IAQ statistical models	ANN, MLR, PLS, DT	✗	✗	✗	✗	✗	✗	✗	✗
[15]	2023	IAQ prediction in schools	ANN, RNN	✗	✗	✗	✗	✗	✗	✗	✗
[17]	2025	Thermal comfort prediction	CNN-LSTM (CFD-based)	✗	✗	✗	✗	✗	✗	✗	✗
[18]	2025	Adaptive IAQ prediction in restaurants	ARX model	✗	✗	✓	✗	✓	✗	✗	✓
[26]	2025	Fungal prediction in public facilities	Gradient Boosting	✗	✗	✗	✗	✗	✗	✗	✗
[19]	2023	Smart environmental control	BPNN + AMOPSO-GWO	✓	✗	✓	✓	✗	✗	✓	✗
[20]	2025	HVAC optimization for schools	MLP + optimization	✗	✗	✓	✓	✗	✗	✗	✗
[21]	2025	AIoT IAQ prediction framework	EPSO, CNN-LSTM	✓	✗	✓	✓	✗	✗	✓	✗
[22]	2023	Secure TinyML deployment	TinyML architectures	✓	✗	✗	✗	✗	✗	✓	✗
[23]	2022	Blockchain-FL for UAVs in B5G	Blockchain, FL	✓	✗	✗	✗	✓	✗	✓	✓
[24]	2025	QoS optimization in IoT-WSN	GWO + Edge Intelligence	✓	✗	✓	✓	✗	✗	✓	✗
[25]	2025	Edge-enabled sustainable transport	Edge Computing + AI	✓	✗	✗	✗	✗	✗	✓	✗
Proposed Model	2025	Real-time IAQ optimization and control	TFT, Bi-LSTM, KD, Fuzzy Logic	✓	✓	✓	✓	✓	✓	✓	✓

**Table 2:** Validation of the proposed CRI against occupant discomfort and IAQ guideline indicators.

Validation Metric	Result	Interpretation
Spearman’s correlation (CRI vs discomfort)	ρ=0.71 (p<0.001)	Strong positive association
Kruskal–Wallis test	p<0.001	Significant group differences
Mean CRI (None)	0.29±0.08	Low discomfort
Mean CRI (Moderate)	0.52±0.11	Moderate discomfort
Mean CRI (High)	0.78±0.10	High discomfort
Mean CRI (${\mathrm{CO}}_{2}\leq 1000$ ppm)	0.35±0.09	Acceptable IAQ condition
Mean CRI (${\mathrm{CO}}_{2}>1000$ ppm)	0.74±0.12	Elevated ventilation risk
Mean CRI (PM_2.5_ ≤ 15 μg/m^3^)	0.38±0.10	Acceptable exposure
Mean CRI (PM_2.5_ > 15 μg/m^3^)	0.76±0.09	Increased health-risk condition

**Table 3:** Output control variables and their corresponding states.

Control Variable	Possible States
Fan speed	Low, Medium, High
Purifier intensity	Off, Low, Medium, High, Very High
Ventilation level	Closed, Partial, Full
HVAC mode	Cool, Maintain, Heat

**Table 4:** Fuzzy membership functions for I/O variables.

Variable	Type	Fuzzy Sets
CRI	Input	Low, Moderate, High
CO_2_ (ppm)	Input	Normal, Elevated, Unhealthy
PM_2.5_ (µg/m^3^)	Input	Good, Moderate, Poor
Humidity (%)	Input	Dry, Comfortable, Humid
Temperature (°C)	Input	Cold, Moderate, Hot
Fan speed	Output	Low, Medium, High
Purifier intensity	Output	Off, Low, Medium, High, Very High
Ventilation level	Output	Closed, Partial, Full
HVAC mode	Output	Cool, Maintain, Heat

**Table 5:** Development environment and hardware configuration.

Component	Specification/Tool
Programming Language	Python 3.10
Deep Learning Framework	PyTorch Lightning (TFT implementation)
Fuzzy Logic Library	Scikit-Fuzzy (custom rule base)
Cloud Training Platform	Google Colab (NVIDIA A100 GPU)
Edge Controller	Raspberry Pi 4B (8GB RAM, Ubuntu 22.04)
IoT Microcontroller	ESP32-WROOM (UART/I^2^C)
Sensors	PMS7003 (PM_2.5_/PM_10_), SCD30 (CO_2_), SDS011 (Outdoor PM)
Communication Protocol	MQTT (sensor-to-gateway and gateway-to-actuator)
Actuators	Wi-Fi smart relays (fan, purifier, HVAC control)
Visualization	Grafana + InfluxDB

**Table 6:** Summary of dataset collected over 30-day deployment.

Property	Description
Deployment Duration	30 Days
Sampling Rate	5 s (sensors), 10 min (APIs), 1 min (final)
Total Samples	43,200 records (1 min aligned) (30 × 24 × 60)
Indoor Features	CO_2_, PM_2.5_, PM_10_, Humidity, Temperature
Outdoor Features	AQI, Temperature, Pressure, Wind Speed
User Feedback	Discomfort labels (None, Moderate, High) via chatbot
Derived Feature	CRI
Ventilation Conditions	Natural and HVAC-assisted
Occupancy Conditions	Variable occupancy activities
Data Format	JSON (key-value pairs per timestamp)
Labeling Type	Soft labels (for fuzzy control simulation)

**Table 7:** Summary of sensing devices and data acquisition specifications.

Device	Measured Parameters	Measurement Range	Accuracy /Uncertainty	Sampling Interval
PMS7003	${\mathrm{PM}}_{2.5}$, ${\mathrm{PM}}_{10}$	0–500 μg/m^3^	±10% (0–100 μg/m^3^)	5 s
SCD30	${\mathrm{CO}}_{2}$, Temperature, Humidity	${\mathrm{CO}}_{2}$ (400–10,000 ppm)	${\mathrm{CO}}_{2}$ (±30 ppm + 3%)	5 s
SDS011	Outdoor ${\mathrm{PM}}_{2.5}$, ${\mathrm{PM}}_{10}$	0–999 μg/m^3^	±15%	5 s
OpenWeather Map API	Temperature, Humidity, Pressure, Wind Speed	Environmental API data	Depends on weather station source	10 min
AirVisual API	Outdoor AQI	AQI index	Depends on monitoring station source	10 min

**Table 8:** Comparison of system performance metrics under baseline and fuzzy control strategies.

Metric	Before Optimization	After Optimization
MAE	0.115	0.078
RMSE	0.182	0.121
MAPE (%)	9.40	5.20
CRRR	0.02	0.27
CPR	0.68	0.91
EEI (kWh)	0.84	0.39
Latency (ms)	575	295

**Table 9:** Ablation analysis of preprocessing impact on forecasting performance.

Dataset Configuration	MAE	RMSE	MAPE (%)
Raw sensor data	0.104	0.168	8.92
Filtered data	0.089	0.142	6.74
Fully processed data	0.078	0.121	5.20

**Table 10:** Intervention efficiency across indoor zones, defined as the ratio of CRI reduction to estimated additional energy usage (ΔCRI/ΔEnergy). Higher values indicate greater relative comfort improvement per unit of estimated energy usage.

Room	HVAC	Window Open	Fan Boost
Living Room	0.72	1.86	0.84
Kitchen	0.63	1.58	0.67
Bedroom	0.76	1.92	0.79
Bathroom	0.84	1.64	0.81
Office	0.78	2.04	0.85

**Table 11:** Comparison of the proposed fuzzy hybrid + optimization model with state-of-the-art predictive models across multiple technical dimensions.

Model	MAE	Latency (ms)	Estimated Actuator EEI (kWh)	Model Size (MB)	Training Time (h)	Interpretability	Fault Resilience
ARIMA	0.106	350	0.83	1.2	0.5	✓	✗
LSTM	0.089	840	0.71	4.3	4.0	✗	✗
GRU	0.085	810	0.68	3.8	3.8	✗	✗
CNN-LSTM	0.081	820	0.70	6.2	5.2	✗	✗
TFT	0.072	960	0.74	12.5	8.5	✗	✗
Informer	0.076	880	0.72	10.4	7.6	✗	✗
Transformer-Encoder	0.079	895	0.73	11.2	8.1	✗	✗
DeepAR	0.084	870	0.75	8.1	6.9	✗	✗
Prophet	0.092	390	0.62	3.1	1.5	✓	✗
DeepState	0.088	800	0.68	9.5	7.2	✗	✗
LightGBM	0.086	470	0.58	2.4	2.3	✓	✗
XGBoost	0.083	450	0.57	2.7	2.6	✓	✗
Proposed Fuzzy Hybrid + Opt.	0.078	295	0.39	2.1	2.3	✓	✓

## Data Availability

The dataset, train/validation/test partitions, preprocessing scripts, and source code supporting the findings of this study are publicly available in an open access repository.
